# Effect of patient sorting done by nurses on care request management in primary care emergency services

**DOI:** 10.17533/udea.iee.v42n3e03

**Published:** 2024-10-11

**Authors:** Genoveva Pérez-Romero, Ángela Jiménez-García, Cesar Hueso-Montoro, Rafael Montoya-Juárez, María Paz García-Caro

**Affiliations:** 1 . Nurse, M.Sc. Email: genoveva.perez.sspa@juntadeandalucia.es. https://orcid.org/0000-0002-8622-3023 Instituto Biosanitario Granada Spain genoveva.perez.sspa@juntadeandalucia.es; 2 . Nurse, M.Sc. Email: angela.jimenez.sspa@juntadeandalucia.es. https://orcid.org/0000-0002-6800-1750 Instituto Biosanitario Granada Spain angela.jimenez.sspa@juntadeandalucia.es; 3 . Nurse, Ph.D. Email: chueso@ujaen.es. Autor de correspondencia. https://orcid.org/0000-0003-1515-3870 Universidad de Jaén Spain chueso@ujaen.es; 4 . Nurse, Ph.D. Email: rmontoya@ugr.es. https://orcid.org/0000-0003-2472-0590 Universidad de Granada Spain rmontoya@ugr.es; 5 . Nurse, Ph.D. Email: mpazgc@ugr.es. https://orcid.org/0000-0002-2763-2572 Universidad de Granada Spain mpazgc@ugr.es; 6 . Servicio de Urgencias de Atención Primaria de Distrito Sanitario Granada Metropolitano. Instituto Biosanitario Granada (Ibs.GRANADA). Granada, España. Instituto Biosanitario Granada Instituto Biosanitario Granada Granada Spain; 7 . Departamento de Enfermería, Universidad de Jaén, Jaén, España. Instituto Biosanitario Granada (Ibs.GRANADA). Centro de Investigación Mente, Cerebro y Comportamiento (CIMCYC), Universidad de Granada. Granada, España. Granada, España. Universidad de Jaén Departamento de Enfermería Universidad de Jaén Jaén Spain; 8 . Departamento de Enfermería, Universidad de Granada. Granada, España. Instituto Biosanitario Granada (Ibs.GRANADA). Centro de Investigación Mente, Cerebro y Comportamiento (CIMCYC), Universidad de Granada. Granada, España. Granada, España. Universidad de Granada Departamento de Enfermería Universidad de Granada Granada Spain

**Keywords:** triage, emergency nursing, advanced practice nursing, community health nursing, primary health care, triaje, enfermería de urgencia, enfermería de práctica avanzada, enfermería de salud comunitaria, atención primaria de salud., triagem, enfermagem em emergência, prática avançada de enfermagem, enfermagem em saúde comunitária, atenção primária à saúde.

## Abstract

**Objective.:**

To determine the influence of patient sorting done by nurses in primary care emergency services on care priorities and discharge referrals, both in general and in relation to the reasons for consultation.

**Methods.:**

Descriptive retrospective study. Variables were compared before and after the involvement of nurses in sorting patients in the primary care emergency services of the Granada Health District (Andalusia, Spain). 41,295 records were analyzed, 18,663 before and 22,632 two years after the inclusion of nurses. The reasons for consultation, priority levels, and types of discharge referral during the two study moments were compared.

**Results.:**

Regarding the reasons for consultation, it was observed that the percentages of malaise (*p<*0.001) and diseases of the genitourinary system (*p<*0.001) increased, while fever (*p<*0.001), among others, decreased. In the two-year measurement period after sorting done by nurses was implemented, type IV priorities increased in percentage (*p<*0.001) and type V priorities decreased (*p<*0.001). Discharges to home decreased (*p*<0.001), while family physician referrals increased (*p*<0.001).

**Conclusion.:**

The participation of nurses in the sorting of patients in primary care emergency services was related to significant changes in priority assignment, discharge referrals, and management of the reasons for consultation, showing an improvement in patient care autonomy and in the resolution of minor clinical problems in the emergency room.

## Resumen

**Objetivo.:**

Determinar la influencia de la enfermera en la clasificación de pacientes de los Servicios de Urgencias de Atención Primaria sobre las prioridades de atención y las derivaciones al alta de manera general y en relación con los motivos de consulta.

**Métodos.:**

Estudio descriptivo retrospectivo. Se compararon las variables antes y después de la inclusión de la enfermera en la clasificación de pacientes de los Servicios de Urgencias de Atención Primaria de la Zona básica de Salud de Granada (Andalucía, España). Se analizaron 41.295 registros, 18.663 antes y 22.632 dos años después de la implementación de la medida. Se compararon los motivos de consulta, niveles de prioridad y derivación al alta en los dos momentos de estudio.

**Resultados:**

En cuanto a los motivos de consulta se pudo observar que, aumentaron los porcentajes de malestar general (*p<*0.001) y enfermedades genito-urinarias (*p<0.001*), y disminuyeron, entre otras, la fiebre (*p<*0.00*1*). En la medición de dos años después de implementada la clasificación por enfermería se incrementaron porcentualmente las prioridades IV (*p<*0.001) y descendieron las prioridades V (*p<*0.001). Las derivaciones al alta al domicilio disminuyeron (*p<*0.001), aumentaron al médico de familia (*p<*0.001).

**Conclusión:**

La participación de la enfermera en la clasificación de pacientes en los Servicios de Urgencias de Atención Primaria se relacionó con cambios significativos en la asignación de las prioridades, las derivaciones al alta, y la gestión de los motivos de consulta evidenciando mejoría en la autonomía de la atención al paciente y en la resolución de problemas clínicos leves de las urgencias.

## Resumo

**Objetivo.:**

Determinar a influência do enfermeiro na classificação dos pacientes dos Serviços de Urgência da Atenção Básica nas prioridades de atendimento e encaminhamentos de alta em geral e em relação aos motivos da consulta.

**Métodos.:**

Estudo descritivo retrospectivo. As variáveis foram comparadas antes e depois da inclusão do enfermeiro na classificação dos pacientes nos Serviços de Urgência de Atenção Primária da Zona Básica de Saúde de Granada (Andaluzia, Espanha). Foram analisados 41.295 registros, 18.663 antes e 22.632 dois anos depois da implementação da medida. Os motivos da consulta, níveis de prioridade e encaminhamento de alta foram comparados nos dois momentos do estudo.

**Resultados.:**

Quanto aos motivos da consulta, observou-se que os percentuais de mal-estar geral (*p<* 0.001) e doenças geniturinárias (*p< 0.001*) aumentaram e a febre, entre outras, diminuiu (*p<* 0.001). Na medição dois anos após a implementação da classificação de enfermagem, as prioridades VI aumentaram percentualmente (*p<* 0.001) e as prioridades V diminuíram (*p<* 0.001). Os encaminhamentos no momento da alta para o domicílio diminuíram (*p<* 0.001) e aumentaram para o médico de família (*p<* 0.001).

**Conclusão:**

A participação do enfermeiro na classificação dos pacientes nos Pronto Socorros da Atenção Básica esteve relacionada a mudanças significativas na atribuição de prioridades, encaminhamentos para alta e gerenciamento dos motivos de consulta, evidenciando melhora na autonomia do atendimento ao paciente e a resolução de problemas clínicos menores em emergências

## Introduction

The increase in emergency care requests by the population is a well-known fact.^1^ In the year 2022 alone, a 30% increase in the number of people that received emergency care in Spain was estimated with respect to the year 2021.^2^ One of the main reasons for this increase has been the rise in the expectations of the population. Users expect to be treated for any alteration in their state of health almost immediately, and to this end a great amount of resources is required.^1^ Previous studies suggest that the population with primary care (PC) difficulties go to the emergency deparment (ED) for this type of care and that between 10% and 60% of the patients in the emergency room could be treated in less urgent care services.^3^^-^^5^

Another factor contributing to the overcrowding of ED is the change in the profile of users. There has been an increase in the age of treated people, an increase in chronicity, complexity, frailty, and comorbidity, which leads to an increase in complications and adverse reactions to medications.^3^^-^^6^ This change in user profile has meant that emergency care, traditionally focused only on the clinical process, has had to adopt a social and family perspective.^6^ Therefore, emergency rooms must make organizational changes to improve the quality and efficiency of care and to adapt it to the users‘ profile.

In recent years, initiatives have been developed to strengthen triage, create new care areas, and modify emergency work processes, which have had positive results, although limited to hospital emergency rooms.^7^^,^^8^ In Andalusia (Spain), a model of emergency care based on integrated and continuous care was adopted, consisting of the hospitals’ emergency services and those of PC centers, as well as the 061 Health Emergency Center.^9^ The Primary Care Emergency Departments (PCED) are located in centers that provide care to the reference population during the closing hours of PC centers (evenings and nights from Monday to Friday, Saturdays, Sundays, and holidays). These services also have mobile teams that provide urgent/emergency care 24 hours a day, providing assisted transfers to referral hospitals when necessary.

To guarantee citizens’ access to the service and to organize healthcare, the PCEDs have incorporated tools such as patient sorting systems at the fixed locations, referral criteria, time-dependent integrated care processes, and clinical process protocols.^10^ In addition, as of 2018, nurses with an advanced practice competency profile (APN)^6^^,^^11^ were incorporated following the competencies recognized for this role in Andalusia.^6^^,^^12^ Specifically, advanced APNs in nursing consultation have specific competencies regarding patient safety in the emergency room, cultural competence, communication and clinical interview, interpersonal relationship skills, management of specific computer systems in the area, and result orientation.^12^

Basic triage by nurses in the ED has been defined as the Reception, Welcome, and Sorting of patients^13^^,^^14^ and is based on the Spanish Triage System with five levels of classification, like its predecessors, the Manchester Triage Scale and the Andorran Triage Model.^15^^,^^16^ Recently, with the boost of APN graduate training, advanced patient sorting or advanced triage is being carried out at the PCEDs, in which APN nurses can refer urgent cases to the reference hospital, offer recommendations and support if the patient has to stay at home, mobilize the corresponding PC team, or resolve the situation immediately and discharge the patient. ^(^^6^ Previous studies in different health systems on the role of APNs or equivalent suggest that they provide better access and reduce waiting times, with similar resource use to that of primary care physicians, including the number of referrals, admissions, revisits and prescriptions, and an even better patient experience.^17^^-^^21^

Despite these results, the impact of nurse presence in patient triage in the PCEDs has not been specifically analyzed. The analysis of some outcome indicators before and after the inclusion of APN nurses competencies in the PCED triage may provide evidence to support the progress of APN nurses in advanced patient sorting and in managing emergency care demandin PC. The objective of this study was to determine the influence of nurses in sorting patients in the PCED though a comparative study of priorities and discharge referrals in relation to the reasons for consultation, before and after the inclusion of nurses in this process in the PCED of Granada (Andalusia) in Spain.

## Methods

A descriptive retrospective study was carried out comparing the emergencies treated in the PCEDs of the Basic Health District of the city of Granada. This district is made up of three PCEDs: La Chana, Zaidín, and Gran Capitán. The areas covered by the different PCEDs are heterogeneous, both in terms of their size and the characteristics of the population they treat; all of them cover city areas and rural localities of the metropolitan area.^22^

The data were obtained from DIRAYA URGENCIAS (DIRAYA-U), which is the health information system of the Andalusian Health Service. The data were provided in an anonymized form and subsequently exported to a database created ad hoc for this study. The database was initially refined by excluding incomplete records concerning the study variables. The years 2017 and 2019 were compared. In 2017, patient sorting was done only by the physician, and in 2018 the incorporation of nurses into patient sorting began, a process that lasted several months, being completed and in force in 2019.

The variables analyzed in the assistance requests were: Age (< 14 years, 15-65, and > 65 years); Sex; PCED (La Chana, Zaidín and Gran Capitán); Type of discharge referral (Admission to another center, Voluntary discharge, To home, To specialist, *Exitus letalis*, Escape, Home hospitalization, Cross-consultation attended, To family physician, To mutual insurance company, Does not attend, Other, Transfer to outpatient care, Transfer to hospital accompanied by medical staff, Transfer to another hospital, Transfer to another service); Priority levels (from I to V) according to the Spanish Triage System; and Reason for consultation (corresponds to the 179 symptoms predetermined in the DIRAYA-U system). To facilitate the analysis, the reasons for consultation were grouped according to ICD-9 coding, although some were treated independently because they fall into various categories of this classification system. For the comparative analysis, only discharges to home, referrals to the family physician, or hospital transfers were considered, since together they accounted for 98.6% and 98.9% of cases in the respective years of study.

The variables level of priority and type of discharge referral and their relationship with the reasons for consultation can be considered indicators of nursing care activity in the sorting and management of care requests. Regarding data analysis, the variables were described by calculating frequency and percentage. To compare the two study years, the Chi-square test or Fisher’s exact test were used for categorical variables, whichever was appropriate. A statistical significance level of 0.05 was considered. The analysis was performed with IBM’s SPSS© v.26 software.

This study was approved by the Andalusian Research Ethics Committee on January 11, 2021, with code EPA_SUAP.

## Results

**Figure 1:**
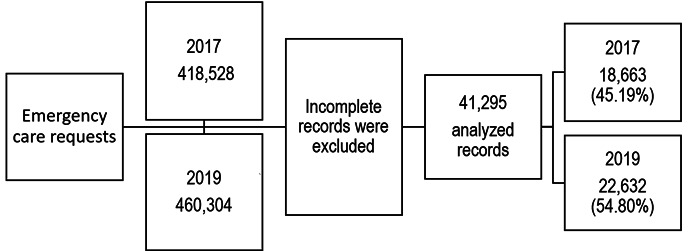
Study scheme

The number of emergency care requests was 418,528 in 2017 and 460,304 in 2019. After excluding incomplete records, 41295 records were analyzed, 18,663 (45.19%) in 2017 and 22,632 (54.80%) in 2019 (Figure 1).

Regarding the characteristics of the analyzed records in both years, an increase of patients in the age range between 15-65 years (*p<0.001*), in the percentage of female patients (*p=0.004*), and in patients being cared for at the PCED La Chana (*p<0.001*) in 2019 with respect to 2017 was observed (Table 1).

**Table 1:** Comparison of the characteristics of the 2017 records (*n*=18663) with those of 2019 (*n*=22632)

Variables	2017 *n* (%)	2019 *n* (%)	***p-*value**
Age			
Less than14 years old	1652 (8.9)	1612 (7.1)	<0.001
15-65 years old	14778 (79.2)	18255 (80.7)	<0.001
66 or older	2233 (12.0)	2765 (12.2)	0.434
Sex			
Male	7594 (40.7)	8892 (39.3)	0.004
Female	11069 (59.3)	13740 (60.7)	0.004
PCED			
La Chana	6791 (36.4)	9520 (42.1)	<0.001
Zaidín Centro	5421 (29.0)	5915 (26.1)	<0.001
Gran Capitán	6451 (34.6)	7197 (31.8)	<0.001

Some of the reasons for consultation have changed percentagewise in 2019 compared to 2017. Noteworthy is the increase in the percentage in 2019 of cases of diseases of the genitourinary system (*p<*0.001) and of malaise (*p<*0.001), and the decrease in the percentage of mental, neurobehavioral, and neurodevelopmental disorders (*p=*0.029), diseases of the circulatory system (*p=*0.024), and fever (*p<*0.001) (Table 2).

**Table 2:** Comparison of reasons for consultation between 2017 (*n*=18663) and 2019 (*n*=22632)

Reason for consultation	2017 *n* (%)	2019 *n* (%)	***p*-value**
Endocrine, Nutritional and Metabolic Diseases	31 (0.2)	46 (0.2)	0.384
Mental, Neurodevelopmental and Neurobehavioral Disorders	689 (3.7)	746 (3.3)	0.029
Diseases of the Nervous System	16 (0.1)	17 (0.1)	0.704
Diseases of the Blood and Blood-Forming Organs and some Immunity Disorders	4 (0.0)	12 (0.1)	0.105
Disorders of the Eye and Adnexa	436 (2.3)	540 (2.4)	0.740
Diseases of the Ear and Mastoid Process	368 (2.0)	459 (2.0)	0.684
Diseases of the Circulatory System	411 (2.2)	427 (1.9)	0.024
Diseases of the Respiratory System	2907 (15.6)	3162 (14.0)	<0.001
Diseases of the Digestive System	2514 (13.5)	2706(12.0)	<0.001
Diseases of the Skin and Subcutaneous Tissue	2152 (11.5)	2768(12.2)	0.029
Diseases of the Musculoskeletal System and Connective Tissue	1993 (10.7)	2158 (9.5)	<0.001
Diseases of the Genitourinary System	1900 (10.2)	2965 (13.1)	<0.001
Complications of Pregnancy, Childbirth, and the Puerperium	5 (0.0)	1 (0.0)	0.098
Injuries, Poisonings and other External Causes	29 (0.2)	36 (0.2)	0.925
Dyspnea	183 (1.0)	210 (0.9)	0.583
Transient loss of Consciousness	1 (0.0)	0 (0.0)	0.452
Dizziness	427 (2.3)	455 (2.0)	0.052
Malaise	2261 (12.1)	3632 (16.0)	<0.001
Tremor	11 (0.1)	4 (0.0)	0.029
Fever	1938 (10.4)	1826 (8.1)	<0.001
Fatigue (not dyspnea) - Asthenia - Weakness - Lack of energy	28 (0.2)	35 (0.2)	0.905
Epistaxis	50 (0.3)	46 (0.2)	0.175
Lump in abdomen	3 (0.0)	5 (0.0)	0.737
Lump in neck	12 (0.1)	17 (0.1)	0.680
Vertigo	19 (0.1)	21 (0.1)	0.769
Headache	275 (1.5)	338 (1.5)	0.867

Regarding the priorities identified in triage, there was a notable increase in the percentage of emergencies identified as priorities IV (*p<0.001*) and a significant decrease in priorities V (*p<*0.001), showing a more distributed allocation of these priorities in 2019 than in 2017. 

The detailed analysis of the relation between priorities and the reasons for consultation showed that, in 2019, there was an increase in cases of malaise classified as Priority II (*p=*0.002), and in cases of diseases of the circulatory system (*p<*0.001), dyspnea (*p*<0.001), malaise (*p<*0.001), and headache (*p<*0.001) classified as Priority III. 

Cases of disorders of the eye and adnexa (*p=*0.031), of diseases of the skin and subcutaneous tissue (*p<*0.001), of diseases of the genitourinary system (*p*<0.001), and of those characterized as malaise (*p<*0.001) classified as Priority IV also increased. Finally, the percentage of cases of diseases of the skin and subcutaneous tissue (*p<*0.00*1*), of diseases of the genitourinary system (*p<*0.001), and of malaise (*p<*0.001), classified as Priority V also increased in 2019 (Table 3).

**Table 3:** Comparison between reasons for consultation according to level of priority (significant data)

Reason for consultation/priority	2017 *n* (%)	2019 *n* (%)	***p*-value**
Priority I (Total)	7 (0.0)	10 (0.0)	0.739
Priority II (Total)	96 (0.5)	116 (0.5)	0.979
Malaise	1 (1.0)	14 (12.1)	0.002
Epistaxis	4 (4.2)	0 (0.0)	0.041
Priority III (Total)	1032 (5.5)	1107 (4.9)	0.004
Diseases of the Circulatory System	26 (2.5)	83 (7.5)	<0.001
Diseases of the Respiratory System	172 (16.7)	112 (10.1)	<0.001
Diseases of the Digestive System	187 (18.1)	139 (12.6)	<0.001
Diseases of the Musculoskeletal and Connective Tissue	133 (12.9)	71 (6.4)	<0.001
Dyspnea	12 (1.2)	44 (4.0)	<0.001
Malaise	82 (7.9)	167 (15.1)	<0.001
Headache	9 (0.9)	35 (3.2)	<0.001
Priority IV (Total)	1515 (8.1)	8989 (39.7)	<0.001
Disorders of the Eye and Adnexa	24 (1.6)	224 (2.5)	0.031
Diseases of the Respiratory System	314 (20.7)	1255 (14.0)	<0.001
Diseases of the Digestive System	224 (14.8)	1095 (12.2)	0.005
Diseases of the Skin and Subcutaneous Tissue	106 (7.0)	906 (10.1)	<0.001
Diseases of the Genitourinary System	160 (10.6)	1370 (15.2)	<0.001
Malaise	179 (11.8)	1556 (17.3)	<0.001
Fever	177 (11.7)	752 (8.4)	<0.001
Fatigue (not dyspnea) - asthenia - weakness - lack of energy	5 (0.3)	9 (0.1)	0.040
Priority V (Total)	16013 (85.8)	12410 (54.8)	<0.001
Diseases of the Circulatory System	342 (2.1)	183 (1.5)	<0.001
Diseases of the Digestive System	2088 (13.0)	1457 (11.7)	0.001
Diseases of the Skin and Subcutaneous Tissue	1955 (12.2)	1757 (14.2)	<0.001
Diseases of the Genitourinary System	1642 (10.3)	1497 (12.1)	<0.001
Dyspnea	144 (0.9)	85 (0.7)	0.045
Malaise	1999 (12.5)	1895 (15.3)	<0.001
Tremor	11 (0.1)	2 (0.0)	0.040
Fever	1644 (10.3)	943 (7.6)	<0.001

Regarding total referrals, home referrals decreased percentagewise in 2019 in comparison to 2017 (*p<*0.001), while family physician referrals increased (*p*<0.001). The detailed referral analysis regarding each of the priorities shows that, in 2019, for Priority III, hospital referrals (*p=*0.002) and home discharges (*p<*0.00*1*) increased, while family physician referrals decreased (*p*<0.001). As for Priority V, the home referral percentage increased in 2019 (81.1%, *p=*0.003), while family physician referrals decreased (*p=*0.011) (Table 4).

**Table 4:** Comparison between priorities and the most prevailing referrals according to level of priority

Priorities		**2017 (*n*=18663)**	**2019 (*n*=22632)**	***p*-value**
TOTAL	Hospital referral	316 (1.7)	416 (1.8)	0.267
	Home referral	14031 (75.2)	16682 (73.7)	<0.001
	Family physician referral	4052 (21.7)	5297 (23.4)	<0.001
Priority I	Hospital referral	6 (85.7)	7 (70.0)	0.452
	Home referral	1 (14.3)	3 (30.0)	0.452
	Family physician referral	0 (0.00)	0 (0.00)	N.A.
Priority II	Hospital referral	25 (26.0)	33 (28.4)	0.696
	Home referral	36 (37.5)	57(49.1)	0.089
	Family physician referral	32 (33.3)	24 (20.7)	0.038
Priority III	Hospital referral	31 (3.0)	64 (5.8)	0.002
	Home referral	243 (23.5)	562 (50.8)	<0.001
	Family physician referral	727 (70.4)	439 (39.7)	<0.001
Priority IV	Hospital referral	20 (1.3)	116 (1.3)	0.925
	Home referral	985 (65.0)	5992 (66.7)	0.210
	Family physician referral	498 (32.9)	2810 (31.3)	0.212
Priority V	Hospital referral	234 (1.5)	196 (1.6)	0.419
	Home referral	12766 (79.7)	10068 (81.1)	0.003
	Family physician referral	2795 (17.5)	2024 (16.3)	0.011

Detailed referral analysis according to the reasons for consultation showed that in 2019 hospital, home, and family physician referrals increased for those patients who went to the emergency room due to malaise; these referrals decreased for patients with fever. Those patients with skin and subcutaneous tissue diseases and genitourinary diseases were discharged to home in a higher percentage in 2019 (*p=*0.008 y *p<*0.001, respectively), and patients with genitourinary diseases were referred to family physicians in a higher percentage in 2019 (Table 5). 

**Table 5:** Comparison of discharge referrals regarding the reason for consultation (significant data)

Reason for consultation/Discharge referral	2017	2019	***p*-value**
Hospital referrals	***n*=316**	***n*=416**	
Malaise	24 (7.6)	62 (14.9)	0.002
Fever	41 (13.0)	27 (6.5)	0.003
Home referrals	***n*=14031**	***n*=16682**	
Mental, Neurodevelopmental and Neurobehavioral disorders	513 (3.7)	535 (3.2)	0.031
Diseases of the Respiratory System	2163 (15.4)	2355 (14.1)	0.001
Diseases of the Digestive System	1735 (12.4)	1874 (11.2)	0.002
Diseases of the Skin and Subcutaneous Tissue	1715 (12.2)	2209 (13.2)	0.008
Diseases of the Musculoskeletal System and Connective Tissue	1469 (10.5)	1599 (9.6)	0.010
Diseases of the Genitourinary System	1507 (10.7)	2172 (13.0)	<0.001
Malaise	1816 (12.9)	2733 (16.4)	<0.001
Fever	1479 (10.5)	1329 (8.0)	<0.001
Family physician referrals	***n*=4052**	***n*=5297**	
Diseases of the Respiratory System	705 (17.4)	760 (14.3)	<0.001
Diseases of the Digestive System	663 (16.4)	708 (13.4)	<0.001
Diseases of the Musculoskeletal System and Connective Tissue	457 (11.3)	498 (9.4)	0.003
Diseases of the Genitourinary System	367 (9.1)	748 (14.1)	<0.001
Dizziness	95 (2.3)	93 (1.8)	0.044
Malaise	394 (9.7)	803 (15.2)	<0.001
Fever	400 (9.9)	449 (8.5)	0.020

## Discussion

In this study, we aimed to determine the influence of nurses when sorting patients in the PCED of Granada by means of a comparative analysis of the levels of priority assigned and discharge referrals regarding the reasons for consultation, before (in 2017) and after the inclusion of nurses in the process (in 2019). In the current international scenario, the roles played by healthcare professionals in primary care are being revised. On this matter, this study provides knowledge for proposing a hypothesis on the role nurses could play in emergency care within primary care.

The first surprising result was the high number of incomplete records in the DIRAYA-U system, which hampered the analysis of over 55% of the records from 2017 and over 45% from 2019. There is abundant literature on the quality of data and the problems in implementing digital records in emergency rooms, in which staff express that there are several problems in completing them, such as staff rotation, pressure to provide care, or excessive bureaucracy.^23^^,^^24^ Other highlighted problems are related to the complex structure of systems intended for the standardization of data (e.g., drop-down menus or restrictions in text input), which can lead to problems caused by system design.^25^ However, it is necessary to point out that in 2019 the percentage of completed records improved noticeably. This can be explained by the inclusion of nurses in the triage record system and the implementation of the PCED. A recent study showed that nurses complete digital records more easily and feel more satisfaction when using them, regarding their collaborative aspects, than doctors, especially in primary care.^26^

Likewise, we found that, when compared to the standard patient sorting management in PCEDs, involving nurses in the process produced significant changes in the allocation of Priorities III, IV, and V, and in discharge referrals to home and to the family physician. We have found that the involvement of nurses has improved the assignment of priority levels in patient care, especially at low complexity levels. This is a result consistent with the innovation that resulted from nurse participation in improving the patient sorting system and broadening he perspective on the emergency itself, which entails including the perspective of care.^12^^,^^27^ This means that, along with seriousness, aspects such as complexity or frailty are considered in the requests of patients, making it possible to distinguish more clearly those problems of lesser clinical concern.^12^^,^^28^ Previous studies have pointed out the excellent quality of the care provided by these professionals, highlighting that the comprehensive emergency care they provide is safe and effective, patient-centered, timely, and efficient.^20^^,^^29^

Discharge decisions, however, seem to have been more poorly resolved with the intervention of nurses, since, overall, there were fewer in situ cases discharged to home and an increase in family physician referrals. However, the analysis of the relationship between Priorities and discharge referrals shows that in Priorities III and V, with nurse intervention, a significantly higher percentage of in situ cases was resolved with home discharge and a lower percentage of family physician referrals. This is consistent with the competencies described in emergency nurse consultation for low-complexity situations.^6^ Several studies reported that nurses, with appropriate training, can manage low-complexity acute health problems with a quality of care comparable to that of general physicians in terms of problem resolution.^18,27)^ Some authors recommend being cautious with this type of results and increasing research when the role of the nurse in triage is not consolidated,^27^^,^^30^ or when there is diversity in the training and experience of the nurses.^31^^,^^32^ It should also be pointed out that in Priority V there was an increase in home referrals, which is relevant since, in addition to improving patient care, it also makes it more adequate and reduces the use of resources.

The analysis of the reasons for consultation in relation to priorities and discharge referrals showed that, compared to standard care, nurse intervention resulted in significant variations in the level of priority assignment of relatively emergent emergency or non-emergent situations (Priority III, IV, and V), as foreseen in the reference document.^6^ Few studies address this perspective and this issue. The literature review by Laurant *et a*l.,^20^ which includes emergency care in PC, although it does not analyze it specifically, suggests that nurse-delivered care, compared to physician-delivered care (in PC), probably results in similar or better health outcomes for a wide range of patient conditions with a low to moderate level of evidence of certainty. Jennings *et a*l.,^33^ who analyzed the care provided by emergency nurses, found that the most common referrals were to home and to the family physician, consistent with our results. In their research they point out that emergency nurses practice a truly hybrid model of service delivery, having both medical and nursing skills with an emphasis on health promotion, education, and comprehensive care.

Consistent with this argument, we consider that the results of the indicators analyzed before and after the nurse intervention in patient sorting in the PCEDs offer some evidence that nurses can be autonomous when administering patient care and resolving minor clinical problems, following the concept of nurse patient request management and the role of the APN,^34^ who, under an agreed protocol and within their scope of competence, carries out advanced patient sorting while managing emergency requests in PC. However, further studies are needed to confirm and broaden these results. It also remains to be determined whether the competencies of the triage nurse in the PCED are developed similarly during periods of high patient visitation.

This study has limitations in generalizing its results since it is an observational retrospective study of one health district and three PCED units. Likewise, it was not possible to control for other variables that may have influenced priority classification and discharge referral, which may not depend on the presence of nurses in the triage process.

We can conclude from this study that these findings provide evidence of the role that nurses can play in emergency care in PC services concerning triage. The involvement of these professionals has been related to the determination of priorities, discharge referrals, and management of the reasons for consultation. This shows that these professionals may have some autonomy in patient care and in the resolution of minor problems in PC emergency settings.
